# Sport as a Factor in Improving Visual Spatial Cognitive Deficits in Patients with Hearing Loss and Chronic Vestibular Deficit

**DOI:** 10.3390/audiolres11020027

**Published:** 2021-06-19

**Authors:** Giorgio Guidetti, Riccardo Guidetti, Silvia Quaglieri

**Affiliations:** 1 Vertigo Center, PCM 41125 Modena, Italy; guidetti.r82@gmail.com; 2 Otorinolaringoiatria, IRCCS Policlinico San Matteo, 27100 Pavia, Italy; silviaquaglieri@libero.it

**Keywords:** hearing loss, vestibular deficit, visual spatial working memory, cognition, Corsi’s test

## Abstract

Hearing loss and chronic vestibular pathologies require brain adaptive mechanisms supported by a cross-modal cortical plasticity. They are often accompanied by cognitive deficits. Spatial memory is a cognitive process responsible for recording information about the spatial environment and spatial orientation. Visual-spatial working memory (VSWM) is a kind of short-term working memory that allows spatial information to be temporarily stored and manipulated. It can be conditioned by hearing loss and also well-compensated chronic vestibular deficit. Vestibular rehabilitation and hearing aid devices or training are able to improve the VSWM. We studied 119 subjects suffering from perinatal or congenital hearing loss, compared with 532 healthy subjects and 404 patients with well-compensated chronic vestibular deficit (CVF). VSWM was evaluated by the eCorsi test. The subjects suffering from chronic hearing loss and/or unilateral or bilateral vestibular deficit showed a VSWM less efficient than healthy people, but much better than those with CVF, suggesting a better multimodal adaptive strategy, probably favored by a cross-modal plasticity which also provides habitual use of lip reading. The sport activity cancels the difference with healthy subjects. It is therefore evident that patients with this type of deficit since childhood should be supported and advised on a sport activity or repeated vestibular stimulation.

## 1. Introduction

Spatial memory is a cognitive process responsible for recording information about the spatial environment and spatial orientation. It enables a person to remember different locations as well as spatial relations between objects and it allows one to remember where an object is in relation to another object.

Spatial working memory (SWM) is a kind of short-term working memory (WM) that allows spatial information to be temporarily stored and manipulated. It has a limited capacity and is quite vulnerable to interference.

It is well known that the dorsolateral prefrontal cortex takes part in SWM thanks to a network, extending across several cortical areas, including the posterior parietal cortex, frontal eye field, supplementary motor area, premotor cortex, anterior cingulate cortex, occipital cortex, and hippocampal formation.

In addition to being closely intertwined attentional and oculomotor programming, in particular related to saccadic movements, it has also been shown that SWM involves higher order cognitive processes, such as executive functioning, at the earliest stages of information processing. Thus, depending on strategies elaborated and task demands, the same spatial information may be represented in SWM by different patterns of activation in the brain.

This view is consistent with a model of memory arising from the interaction between higher-order cognitive top-down processes governed by the prefrontal cortex and stimulus-specific brain regions. Visual attention has a cross-modal influence on activity in this network. The visual spatial exploration component provides information for the visual spatial working memory (VSWM).

Spatial working memory problems are frequently reported following brain damage within both left and right hemispheres but with the severity often being greater in individuals with right hemisphere lesions.

The increase in performance with advancing age supports the notion that SWM capacity increases with maturation throughout childhood with an ameliorative effect of education. It declines across the life span even in the absence of disease-related cerebral pathology.

Sex differences are often reported in spatial abilities. Until a few years ago, it was widely accepted that men outperformed women on almost all spatial tasks. However, some studies show conflicting results, which can be ascribed to the complexity of the variables involved in the visuo-spatial domain, and can be better explained by differences in spatial competences. Indeed, these differences could reflect the use of different strategies, rather than different competences, used by the two sexes.

Hearing loss and chronic vestibular pathologies require adaptive cerebral mechanisms capable of modifying the usual networks, they can interfere between themselves and thus, they are often accompanied by cognitive deficits which become clearer with aging [1,2,3].

In both pathological conditions, the adaptation is supported by a cross-modal cortical plasticity. Cross-modal plasticity refers to the phenomenon when deprivation in one sensory modality (e.g., the auditory modality as in deafness or vestibular deficit) results in the recruitment of cortical resources of the deprived modality by intact sensory modalities (e.g., visual or somatosensory systems) [4,5,6].

In particular, the adaptation to vestibular deficit is considered an important example of this kind of neuronal plasticity. Thanks to wide central connections, the vestibular system is not merely involved in reflexes, but it is also connected to cognitive processes. A growing body of literature suggests that it has a substantial impact on cognitive function. These cognitive interactions include memory, attention, mental imagery, body awareness, and social cognition. Emerging research suggests that the vestibular system can be considered a potential window for exploring brain function beyond that of maintenance of balance, and into areas of cognitive, affective and psychiatric symptomology [7].

Cognitive deficits occur frequently among patients with vestibular abnormalities of any type [8]. Behavioral studies in rodents and humans have demonstrated that damage to the vestibular system specifically leads to cognitive deficits in spatial learning and memory, navigation, mental rotation, and mental representation of three-dimensional space, which are not necessarily related to any particular episode of vertigo or dizziness, and therefore these deficits may occur even in patients who are otherwise well-compensated [8,9,10,11,12,13], especially so for the elderly [14]. Neither the side of the lesion nor the duration of disease influences cognitive performance. SWM deficits are usually not associated with general memory deficits or whole brain atrophy.

Children with VL show similar cognitive difficulties to adults, in tasks involving dynamic cognitive processes (higher attentional load) that in tasks requiring static cognitive processes such as visual attention task [15].

In addition, the relationship between peripheral hearing loss and cognition are well-documented in previous comprehensive reviews [16,17].

Particularly in mice, even only a moderate hearing loss is characterized by progressively poorer performances in spatial working and recognition memories, with more p-tau and lipofuscin in the hippocampus [18,19].

It has been speculated that hearing loss was associated with an increased rate of dementia diagnosed before age 60 [20], and that vestibular loss can facilitate mild cognitive impairment and Alzheimer’s disease [21].

Hearing loss in children leads to various deficiencies, such as an impaired language, poorly developed reading and writing skills, difficulties in mnemonic learning, deficit in mathematical reasoning, and an inability to comprehend space and time.

Hearing loss which arises in infancy can be considered a substantial signal of weakness (a non-specific state of vulnerability, a reduced physiological reserve and a reduced resistance to stress) especially with aging; this leads to a deterioration in memory, perception, attention and linguistics.

This condition, especially in children, represents a social isolation risk and a reduced capability to participate in social activities such as sports.

Regarding this, it is worth mentioning that in Geneva in 1992 UNESCO (UNESCO, Service des loisirs, Geneva, Switzerland, 1992) redacted the paper for the children’s rights in sport, and in 11 points it underlines that sport is a fundamental right for children and a commitment to exercise helps them to release tension and grow up healthy and fit.

In particular, the paper underlines that parents must encourage physical exercise due to the notorious psychophysics advantages which cannot be regained in a later age, so parents must not deny children those possibilities.

Severe hearing and vestibular deficits are often associated with patients with newborn and infant hearing loss. The balance training proved effective in improving SWM in healthy people [22,23,24]. Vestibular rehabilitation [25] and auditory and cognitive training [26,27] proved effective in improving the SWM, and also in-patient suffering from chronic vestibular deficit and hearing loss.

Hearing devices (cochlear implants and bone-anchored hearing implant) can improve WM in children with sensorineural hearing loss [5,28].

In particular, children with cochlear implants demonstrated better performance in VSWM and short-term memory skills than in auditory working memory and auditory short-term memory skills. Significant positive relationships were found between visual working memory and reading outcomes [29].

There is a lack of studies on the possibility that sport is able to interfere with any cognitive problems connected with the simultaneous hearing and vestibular deficit. We therefore considered the possibility of using VSWM to evaluate the ability of sports activity to improve cognitive functions.

## 2. Methods

Collaborating with the scientific board of Ente Nazionale Sordi (ENS), specifically with the Milan and Reggio Emilia branches, and with the Federazione Sport Sordi Italia (FSSI) we examined 104 deaf individuals, divided into two groups; those over and those under 65 years old.

The sample (ENS group) was composed of 77 men (74%) and 27 women (26%). The average age was 49 years old (SD 18). Participation was exclusively voluntary.

Bilateral hearing loss was always present, severe in 26 individuals (25%) and profound in 78 (75%). The hearing loss was present at birth in 61 cases (58.6%), within the first year in 34 (32.8%), and within 13 years in the remaining 9 (8.6%).

Hearing loss was hereditary in 41 cases (39.4%). The cause was unknown in 32 (30.8%), post-infective in 31 (29.8%).

No individual had a cochlear implant.

All the subjects examined communicate using correct sign language and lipreading.

Only 32 subjects (30.8%) had had vertigo during their lifetime.

Of the participants, 73 (70.2%) regularly practiced various sports (FSSI group), 31 (29.8%) never practiced physical activity, or practiced irregularly (ENS group).

An additional group examined was the Italian national deaf female volleyball team (TEAM volley group) which was the silver medal at the 2018 Paralympics and the 2019 European Championship.

The group is composed by 15 athletes with an average age of 22.7 years old (SD 3.99). In this group the cause of deafness is hereditary in 7 cases (46.7%), unknown in 5 (33.3%), and viral in 3 (20%).

Hearing loss originated at birth in 12 cases (80%), within the first few months in 2 (13.3%) and within 3 years in one case (6.7%). Three athletes had a cochlear implant. Only one athlete had had vertigo.

Thanks to the translation of sign language, every individual was adequately and preemptively instructed on the tests they would being subject to, for both the methods and the scope of the test.

**Figure 1:**
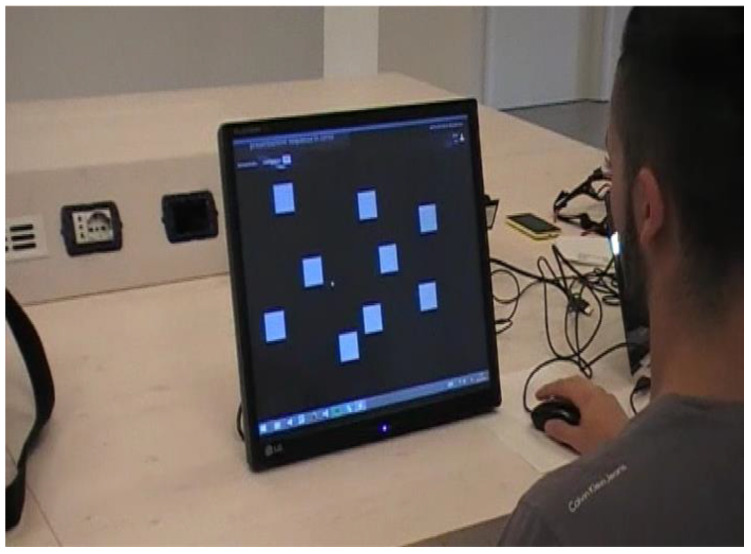
The digital version of Corsi’s test (eCorsi test).

The occurrence of a vestibular deficit was evaluated through the Video Head Impulse Test (v-IHT) [30,31,32]. The patient is sitting and is looking forward, she is asked to keep her eyes fixed on a target positioned on a wall about a meter away. The examiner who is behind the head of the patient, keeping it still, rotates it abruptly and unpredictably towards the left or the right with an amplitude of a maximum of 10–20 degrees for 20 times per side. The head and eye movement are recorded by goggles worn by the patient which are equipped with cameras. If the examined labyrinth, and therefore her vestibulo-ocular reflex (VOR), is normal, the subject is capable of compensating the accelerating head movement stimulated by the examiner and of keeping her gaze on the target. If the VOR gain is pathological, the eyes lose track of the target during the head’s rotational movement as they rotate exactly in the same direction and with same speed. The pathological subject, differently from the normal subject (Figure 1), at the end of the abrupt rotation will make a corrective rapid ocular movement (known as saccadic) to re-establish the sight on the target. In this manner, the vestibular function of both the labyrinths can be precisely evaluated in less than 10 min. A specific software evaluates the presence of saccadic movements and the VOR gain for each labyrinth. The calibration is rapid and simple, with two lasers built into the goggles.

The single most important nonverbal task for the assessment of visuo-spatial working memory (VSWM) is the Corsi block tapping task (CBTT), also known as the Corsi Span Test. It also involves spatial attention. The traditional version of Corsi apparatus consists of a set of nine identical blocks (3 × 3 × 3 cm^2^) irregularly positioned on a wooden board (23 × 28 cm^2^). The experimenter points to a series of blocks at a rate of one block per second. Subsequently, the participant is required to point to the same blocks in their order of presentation. The length of the block sequences (starting from 2-block sequences) increases by one item until recall is no longer correct. The procedure ends when the number of wrong reproductions exceeds the proportion of admissible errors per length. A span score was calculated corresponding to the larger sequence the subject can correctly reproduce. The maximum score possible is 9.

This test was used mainly in neurological diseases (Alzheimer’s disease, autism spectrum disorder, depression and affective disorders, Down’s syndrome, e, multiple sclerosis, Parkinson’s disease, schizophrenia, stroke and cerebrovascular disease, traumatic brain injury) and it is significantly impaired in patients suffering from chronic peripheral vestibular hypofunction when compared with healthy controls [8,10].

It was also used in sports athletes with a history of concussion [33].

We used the digital version of Corsi’s test (eCorsi test) [34,35] (Figure 1).

In Corsi block-tapping task for digital tablets (eCorsi), instead of cubes to be tapped on a board, the setup consists of squares that flash on a computer screen. Participants reproduce the sequences by tapping blocks on a (touch) screen, without substantial differences between the two versions in terms of performance.

In addition, in the eCorsi test, the final score corresponds to the maximum number of targets reproduced correctly and therefore ranges from a minimum of 2 to a maximum of 9.

The data was confronted with the following:

- 430 subjects without vestibular deficits and hearing loss (NORM); 224 females (52.1%) and 206 M (47.9%), 300 aged 17 to 64 years (mean 31.57, SD 12.41) and 130 aged 65 years or more (mean 72.2, SD 4.79).
- 404 subjects with chronical vestibular failure (CVF group); 210 females (51.9) and 194 males (48.1%), 234 aged 17 to 64 years (mean 48.94, SD 10.44) and 170 aged 65 years or more (mean 75.08, SD 5.25).
- 34 subjects without vestibular deficits and hearing loss who practice volleyball, basketball and football at an amateur level (SPORT group); 14 females and 31 males, with an average age of 25.44 years (SD 4.63).
- 50 professional athletes (PROF group) in basketball, volleyball, motorsport; 15 females and 35 males, with an average age of 23.94 years (SD 3.46) without vestibular deficits and hearing loss
- 18 females NORM who do not practice sport (NORM NS group) and 25 who often practice volleyball (NORM V group) at a professional level on par with the Italian national deaf volleyball team, without vestibular deficits and hearing loss. The average age was equivalent to the TEAM volley’s group.

Statistical analysis was performed by Statistical Package for Social Science (SPSS).

Groups were tested for normality. The difference between the means of normally distributed variables was calculated using Student’s *t*-test. A paired sample *t*-test was performed to assess the differences in the same subject, while an independent sample *t*-test was used to compare two groups. Differences with a *p*-value < 0.05 were considered statistically significant.

## 3. Results

A bilateral vestibular deficit was present in 66 subjects (63.5%, ENS VF group) and in 38 (36.5%, ENS noVF group) it was either absent or non-significant.

In 31.8% of subjects with vestibular deficit, vertigo was present in the medical history and in 28.2% of subjects without a clear vestibular deficit. Therefore, all cases are to be considered in a state of good vestibular system adaptability.

Both CVF and ENS have a statistically lower score than NORM (*p* < 0.0001), as shown in Table 1. Previous studies showed that the score difference between subjects with unilateral and bilateral CVF is not significant (*p* = 0.0734) [25].

In ENS, the presence of a vestibular deficit does not significantly change the score (*p* = 0.6921).

ENS have a better score than CVF (*p* = 0.0009).

ENS NS have an equivalent score to the CVF group (*p* = 0.9113).

FSSI score does not differ from the NORM group (*p* = 0.0928).

ENS NS have a significantly lower score than NORM (*t*-test: *p* < 0.0001).

FSSI have score values equivalent to those of NORM (*t*-test: *p* < 0.0928).

In subjects over 65, the difference between ENS and NORM is lower (*p* = 0.0026) and is significant between the ENS itself and CVF (*p* = 0.0154) and between CVF and NORM (*p* < 0.0001).

FSSI have a significantly better score than NORM for the same age (*p* = 0.0130).

The comparison between the score of 34 SPORT and 34 FSSI with the same athletic skill level shows (Table 2) a non-significant difference, even if the FSSI group’s age is relatively larger (*p* = 0.0005).

The score of both groups is lower than that of the PROF group. The comparison between SPORT and PROF, with not quite statistically significant age difference, confirmed a statistically better performance in PROF.

In the group containing the 15 athletes of the Italian national deaf volleyball team (TEAM volley group), eight had a vestibular deficit, four unilateral and four bilateral.

In comparison (Table 3), 18 subjects in NORM who do not practice sport (NORM NS group) and 25 who regularly practice volleyball (NORM V group) with the same gender and age showed a significantly better score (*t*-test: *p* < 0.0011) in those who practice sport rather than those who do not; the athletes of the national team have a score equivalent to the score of the athletes with normal hearing (*t*-test: *p* = 0.8857).

In the totality of ENS and TEAM volley, there is a small but significant male advantage (Table 4).

However, the difference is inferior to the one observed in NORM (Table 5). Comparing the TEAM volley score with the 34 FSSI with the same athletic skill level, the difference vanishes (*p* = 0.4641).

In CVF, the difference is not significant (Table 6).

## 4. Discussion

The subjects suffering from chronic hearing loss and/or unilateral or bilateral vestibular deficit have shown a VSWM less efficient than that of the NORM group.

The contemporaneous presence of these two deficits does not seem to significantly change this cognitive function.

However, on average, ENS have a better score than CVF; this hints at a better adaptive strategy, likely supported by a cross-model plasticity which includes the regular use of lipreading [36].

This characteristic occurs mainly in subjects who practice sport: their VSWM is equivalent to NORM’s, or even better for subjects in the over 65 age group.

The effect of physical activity is undeniable for the same athletic skill level; the performance difference with respect to NORM vanishes for amateurs and professional athletes.

Therefore, it appears to be confirmed that motor skill learning induces some differences in brain network plasticity regarding non-active peers [24,37,38].

The comparison between males and females reveals a small but significant male advantage on average, as it was often observed in VSWM tests [25,39,40] after adolescence.

However, the difference is lower than the one found in the NORM, so the likely influence of similar adaptive strategies between the two genders in day-to-day life is confirmed.

Indeed, the difference between genders vanishes completely if we compare the score of TEAM volley and the score of 34 FSSI at the same athletic skill level. This is most likely the effect of a similar and prolonged training.

Indeed, the sex difference in human SWM can be attributed to the complexity of the variables involved in the visuo-spatial domain, and can be better explained by differences in spatial competences. This could reflect the use of different strategies, rather than different competences, in both sexes.

## 5. Conclusions

We have already demonstrated how vestibular rehabilitation is capable of significantly improving the spatial memory of subjects suffering from monolateral or bilateral vestibular deficit.

The study underlines that physical activity, when practiced regularly, allows the same results to be achieved in subjects with profound hearing loss, regardless of vestibular deficit, where VSWM is lower on average than healthy peers.

The study shows that, in both genders, physical activity improves these cognitive functions [37,41,42], promoting the same cross-modal plasticity mechanisms which support the functional adjustment in hearing loss and chronic vestibular deficit.

Thus, it is clear that, in patients with this deficit since infancy, it is suggested to support and recommend physical activity.

When the patient is a newborn, we suggest stimulating the vestibular and cognitive function throughout daily exercises such as “hide and seek”, walking, and flips from 18 months old [43,44].

Indeed, in this way, the newborn can develop spatial-temporal reasoning, a sense of direction, saccadic spatial exploration and motor coordination. Physical activity activates different neural networks compared to a sedentary life; this promotes the development of new cognitive strategies characterized by better spatial-temporal reasoning and navigation skills, which are useful in a person’s social life, in particular in sport and driving [45,46].

## Figures and Tables

**Table 1:** Average values and standard deviations for the Corsi’s test score in the various groups.

Group	Number	Mean	SD
NORM	430	6.181	0.0991
<65 years	300	6.58	0.778
>65 years	130	5.294	0.774
CVF	404	4.732	2.720
<65 years	234	5.184	3.387
>65 years	170	4.112	1.074
ENS	104	5.653	1.453
<65 years	78	5.935	1.332
>65 years	26	4.692	1.435
FSSI	73	6.068	1.377
ENS NS	31	4.677	1.136
ENS VF	66	5.697	1.435
ENS noVF	38	5.579	1.5

**Table 2:** Average values and standard deviations for the Corsi’s test score in NORM and FSSI with equivalent athletic skill level.

Group	Number	Age	Mean	SD
FSSI	34	30.558 (SD 6.679)	6.714	1.045
SPORT	34	25.44 (SD 4.637)	6.72	0.678
PROF	50	23.94 (SD 3.460)	7.28	1.088718
*t*-test FSSI/SPORT	*p* 0.0005		*p* 0.9777	
*t*-testSPORT/PROF	*p* 0.0596		*p* 0.0001	

**Table 3:** Average values and standard deviations for the Corsi’s test score in NORM NS, NORM volley and TEAM volley with the same age and the same athletic skill level.

Group	Number	Mean	SD
NORM NS	18	5.611	1.289
NORM Volley	25	6.51	1.157
TEAM Volley	15	6.46	1.25

**Table 4:** Average values and standard deviation for the Corsi’s test score in ENS and TEAM volley, with respect to gender.

Gender	Number	Means	SD
M	77	6.090	1.273
F	42	5.404	1.531
*t*-test	*p* 0.0123		

**Table 5:** Average values and standard deviation for the Corsi’s test score in NORM, with respect to gender.

Gender	Number	Means	SD
M	206	6.373	0.921
F	224	6.061	0.991
*t*-test	*p* 0.0008		

**Table 6:** Average values and standard deviations for the Corsi’s test score in CVF, with respect to gender.

Gender	Number	Means	SD
M	194	4.804	1.226
F	210	4.666	3.587
*t*-test	*p* 0.6110		

## Data Availability

Data available on request due to restrictions e privacy or ethical. The data presented in this study are available on request from the corresponding author. The data are not publicly available due to patients health data.
